# Corpus Callosum Morphology in Capuchin Monkeys Is Influenced by Sex and Handedness

**DOI:** 10.1371/journal.pone.0000792

**Published:** 2007-08-29

**Authors:** Kimberley A. Phillips, Chet C. Sherwood, Alayna L. Lilak

**Affiliations:** 1 Department of Psychology, Hiram College, Hiram, Ohio, United States of America; 2 Department of Anthropology, The George Washington University, Washington, D. C., United States of America; University of Maryland, United States of America

## Abstract

Sex differences have been reported in both overall corpus callosum area and its regional subdivisions in humans. Some have suggested this reflects a unique adaptation in humans, as similar sex differences in corpus callosum morphology have not been reported in any other species of primate examined to date. Furthermore, an association between various measurements of corpus callosum morphology and handedness has been found in humans and chimpanzees. In the current study, we report measurements of corpus callosum cross-sectional area from midsagittal MR images collected *in vivo* from 14 adult capuchin monkeys, 9 of which were also characterized for hand preference on a coordinated bimanual task. Adult females were found to have a significantly larger corpus callosum: brain volume ratio, rostral body, posterior midbody, isthmus, and splenium than adult males. Left-handed individuals had a larger relative overall corpus callosum area than did right-handed individuals. Additionally, a significant sex and handedness interaction was found for anterior midbody, with right-handed males having a significantly smaller area than right-handed females. These results suggest that sex and handedness influences on corpus callosum morphology are not restricted to *Homo sapiens*.

## Introduction

The corpus callosum (CC) is the major white matter tract connecting the left and right cerebral hemispheres, with fibers establishing both homotopic and heterotopic connections along an anterior-posterior gradient. Cross-species data from anthropoid primates suggests that reduced interhemipsheric connectivity via the CC is related to the enhancement of structural asymmetries [1]. Whether differences among individuals in the size and/or shape of the CC and its subdivisions within a species exist as a function of sex, age and handedness has been the subject of considerable controversy.

Sex differences in both overall CC area and its regional subdivisions in humans were first reported by De Lacoste-Utamsing and Holloway [2]. In a departure from earlier studies addressing this issue, De Lacoste-Utamsing and Holloway statistically controlled for total brain size and concluded that women have a larger midsagittal area of the CC and a more bulbous splenium. Numerous studies have since followed, with some replicating the findings of de Lacoste-Utamsing and Holloway [3]–[9] and others not [*e.g*. 10]. These conflicting results are due in part to the limitations of previous methodologies and unstandardized reporting of corpus callosum measures–some adjust for brain size while others do not. Similar sex differences in CC morphology have not been reported in any nonhuman primate species examined to date, including chimpanzees, Old World and New World monkeys [11]–[14], leading some to conclude that these sex differences reflect a unique adaptation in humans [15].

Witelson [16], [17] first proposed that handedness and CC size were related. Her studies have shown that non-consistently right-handed men have larger posterior CC areas than do consistently right-handed men and this difference is present in the anterior and posterior halves of the CC but not in the splenium alone. Since her initial reports, several studies have reported an association between various measurements of corpus callosum morphology and handedness in humans [*e.g*., 8].

Whether other primates show similar patterns of sex and handedness influences on CC morphology would enhance our understanding of the neurobiological substrates of handedness, as only a few studies have investigated neural correlates associated with hand preference in nonhuman primates. For example, evidence from chimpanzees indicates that hand preferences for non-communicative actions are correlated with asymmetries of the hand knob region of the precentral gyrus, but not language area homologues [18]. Similarly, asymmetries of the dorsal portion of the precentral gyrus are associated with left-hand preference in male capuchin monkeys [19]. A recent comparative study on chimpanzees and capuchins concluded cerebellar asymmetries were significantly associated with handedness and this effect was most pronounced in right-handed capuchins [20]. To our knowledge, only one study has investigated both behavioral lateralization and CC morphology in nonhuman primates. This study found relationships between corpus callosum morphology and handedness in chimpanzees, with left-handed chimpanzees having several corpus callosum subdivisions (rostrum, anterior midbody, posterior midbody, isthmus and splenium) significantly smaller than right-handed chimpanzees [11]. No sex differences in CC morphology were reported. All together, these limited data indicate that although neuroanatomical asymmetries associated with lateralized behavior are found among some primate species, the relationship between direction of the asymmetry and handedness may be variable across phylogeny.

Here we investigate sex and handedness influences on corpus callosum morphology in capuchin monkeys. The prevailing view is that skilled motor actions are dependent upon left-hemisphere specialization [21]. It has been hypothesized that complex foraging skills, such as tool use and extractive foraging, in hominoids may have driven the selection for lateralization of specific motor behavior [22]. Indeed, population-level biases in hand usage for a variety of tasks have been shown in the great apes [23]–[25]. Because capuchins have convergently evolved a similar degree of complex foraging behavior, they may also show neuroanatomical lateralization. Thus, research on capuchins would add important information to questions pertaining to the neurobiology of handedness for several reasons. First, capuchins are noted for their high degree of manipulative propensities and extractive foraging habits, which are analogous to complex manipulative skills demonstrated by humans and chimpanzees [26]. Second, individual capuchins express strong and consistent hand preferences during tasks that require complex bimanual coordination [27]–[32]. Whether or not capuchins express a tendency towards population-level right-handedness is not clear, with some research groups reporting population-level preferences [*e.g*., 29] and others not [*e.g*., 32], [33]. Given these characteristics and recent findings of neuroanatomical asymmetries and their relationship to lateralized behavior in capuchins, we hypothesized that overall CC midsagittal area and regional subdivisions of the midbody, isthmus, and splenium would be related to handedness in capuchins. Furthermore, we hypothesized that sex effects would not be present because they have not been observed in any previous study of nonhuman primates.

## Methods

### Subjects

*In vivo* magnetic resonance images were collected from 18 capuchin monkeys (*Cebus apella*; male *n* = 10, female *n* = 8) and behavioral data on handedness was collected from 13 (male *n* = 7, female *n* = 6) of these subjects. Ages ranged from 1–21 years (*M* = 10.08±6.65). Of the total subjects, 14 were adults (≥5 years; male *n* = 6, female *n* = 8) and four were juveniles (between 1–4 years; see Tables 1 and 2). Subjects were housed at Hiram College (Hiram, Ohio), Northeastern Ohio Universities College of Medicine (Rootstown, Ohio), the College of Wooster (Wooster, Ohio), or the University of Pittsburgh (Pittsburgh, Pennsylvania). The MRI scanning protocol was approved by the Institutional Animal Care and Use Committee at each of these institutions.

**Table 1:** Unadjusted midsagittal area measures of the CC and its subdivisions, total brain volume, and body weight for each subject.

Subject	Sex	Rostrum	Genu	Rostral body	Anterior midbody	Posterior midbody	Isthmus	Splenium	Total CC Area(mm^2^)	Brain Volume(cc)	Body Weight (kg)
Alou	M	5	12	10.25	7.5	5.25	4	15.5	62.50	77.06	2.24
Carlos	M	2.75	16.75	17	14.5	11.25	10.25	16.5	88.50	98.81	3.96
DiMaggio	M	1.75	16	12.25	9	8	5	14.25	67.75	82.03	1.27
Miro	M	2	12	12.25	5.75	6.75	6.25	14.75	59.75	81.89	6.24
Sabro	M	2	11	12.25	8.50	6.50	7.50	15.00	60.50	64.60	2.60
Shiro	M	1.90	11.25	7.90	6.80	5.40	6.10	14.75	47.90	63.90	2.30
Shoeless	M	2.25	14.25	11.75	7.5	9	5.5	15	66.00	86.35	1.94
Sosa	M	1.25	19	10.25	8.75	7.75	5.25	13.5	60.75	86.51	2.38
Vincent	M	2.75	21.25	9	11.25	7.5	3.75	11.25	66.75	87.83	4.37
M21-02	M	2.5	11	9.25	6	7.25	6.5	16.00	52.50	64.60	3.20
DC	F	3.25	8.25	11.5	10.25	8.75	6.75	19.5	67.50	61.84	2.95
Georgia	F	2	15.75	18	10.75	8	8.5	13.75	72.75	68.82	2.72
Gizmo	F	1.75	13	13.5	10.5	9.75	9.75	16	75.50	63.07	2.73
Jake	F	1.75	13.75	14.5	9.25	9.25	8.75	18.25	78.25	64.72	2.73
LC	F	1.75	14	14.75	8.75	11	8.25	17.25	74.00	55.67	2.35
Noel	F	4	20.75	10.5	8.25	6.5	8.25	16.5	75.50	65.57	2.50
M57-04	F	2	11.5	12.00	7.50	9.25	6.75	16.5	59.30	61.20	2.50
M58-04	F	2.25	13.75	14.75	10.25	11.00	9.25	18.50	83.30	79.50	2.50

**Table 2:** Midsagittal area measures of the CC and its subdivisions (statistically adjusted by dividing the square root of the CC area by the cube root of total brain volume to bring all measures into the same geometric dimensionality), mean handedness index (MHI) for the tube task, and dextral classification.

Subject	Sex	Age	Overall CC	Rostrum	Genu	Rostral body	Anterior midbody	Posterior midbody	Isthmus	Splenium	*M*HI	Dextral Group
Alou	M	2.5	1.86	.525	.812	.751	.643	.538	.470	.924	0.81	R
Carlos	M	5	2.04	.359	.885	.892	.824	.726	.693	.880	−0.95	L
DiMaggio	M	1	1.89	.304	.920	.805	.690	.650	.514	.868	0.39	R
Miro	M	12	1.78	.326	.797	.807	.553	.599	.576	.885	1.00	R
Sabro	M	5	1.94	.353	.827	.873	.727	.636	.683	.966		
Shiro	M	5	1.73	.345	.839	.703	.652	.581	.617	.960		
Shoeless	M	1.5	1.84	.339	.853	.776	.620	.679	.531	.876	−0.14	A
Sosa	M	3.5	1.76	.253	.986	.724	.670	.630	.518	.831	−0.62	L
Vincent	M	18	1.84	.373	1.040	.674	.754	.615	.435	.754	−1.00	L
M21-02	M	7	1.81	.394	.827	.758	.611	.671	.636	.998		
DC	F	21	2.08	.456	.727	.858	.811	.749	.658	1.118	0.96	R
Georgia	F	6	2.08	.345	.968	1.034	.800	.690	.711	.904	−0.75	L
Gizmo	F	16	2.18	.332	.907	.921	.814	.785	.785	1.005	0.60	R
Jake	F	15	2.21	.330	.925	.950	.758	.758	.738	1.065	1.00	R
LC	F	15	2.25	.346	.979	1.005	.774	.868	.752	1.087	0.85	R
Noel	F	14	2.16	.496	1.132	.804	.713	.633	.713	1.008	−0.82	L
M57-04	F	13	1.95	.359	.861	.879	.695	.772	.659	1.031		
M58-04	F	20	2.12	.349	.862	.893	.745	.771	.707	1.000		

Adults are ≥5 years.

### MRI Procedure and Image Quantification Method

Capuchins were transported to the Brain Imaging Research Center in Pittsburgh, Pennsylvania for the MR procedure. Once at the facility, subjects were initially immobilized by ketamine injection (25 mg/kg) and acetylpromazine (1 mg/kg), and subsequently anaesthetized with propofol (160–330 micrograms/kg/minute). Subjects were placed into the scanner chamber and their heads were fitted inside a 16 cm head coil. Subjects remained anaesthetized throughout the MR procedure and respiration rate, heart rate, and oxygen consumption were continually monitored. T1-weighted images were acquired on a 3.0 T scanner (Siemens Allegra). Images were collected in the sagittal plane using a gradient echo protocol (pulse repetition = 1500 ms, echo time = 3.04 ms, and a 256×256 matrix). Subjects were allowed to completely recover from the effects of the anaesthesia before return transport.

**Figure 1:**
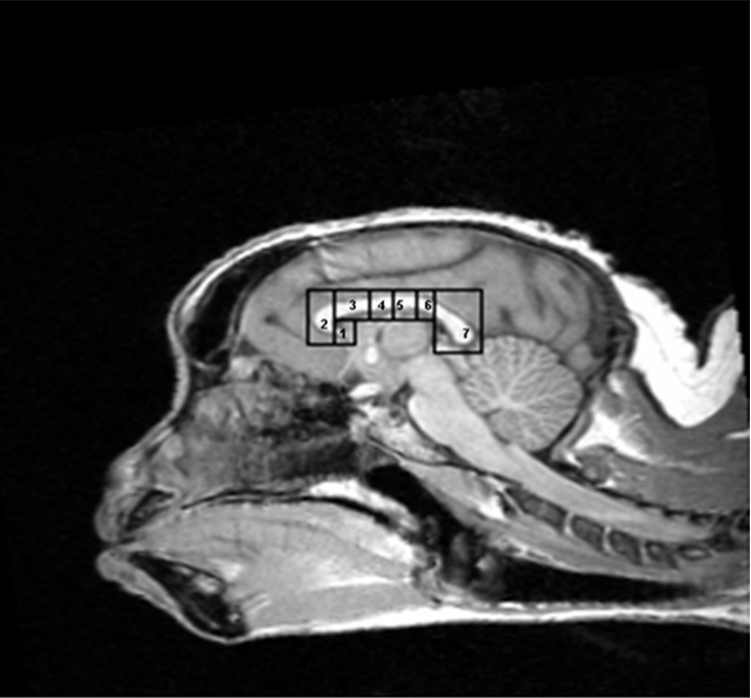
Midsagittal view of capuchin corpus callosum, showing 7 regional subdivisions. These subdivisions are: 1 = rostrum; 2 = genu; 3 = rostral body; 4 = anterior midbody; 5 = posterior midbody; 6 = isthmus; 7 = splenium.

Morphometric measurements of the CC were performed using ImageJ software version 1.26t (http://rsb.info.nih.gov/ij/) and followed the methodology of Witelson [7]. The midsagittal area of the CC was measured in its entirety. Seven subdivisions of the CC were defined and can be seen in Figure 1. To subdivide the CC, first the entire length of the CC was measured, and divided into thirds. The anterior third was further divided into three regions by tracing a vertical line through the point where the anterior CC began to curve back slightly. This resulted in three subdivisions: rostrum (1), genu (2), and the rostral body (3). The middle third of the overall CC was subdivided into equal sections, resulting in the anterior midbody (4) and posterior midbody (5). Finally, the posterior third of the overall CC was subdivided into the isthmus (6) and splenium (7). The splenium was defined as the posterior fifth of the entire CC; the remaining area within the posterior third was defined as the isthmus.

### Behavioral Measures

Hand preference was determined through a coordinated bimanual task known as the tube task [34]. This task was chosen because it elicits a high degree of hand preference in nonhuman primates and it is stable within an individual over time [35]. Although different conclusions have been reached with respect to whether this task does [29] or does not [32] elicit population-level hand preferences in capuchins, it is clear that individuals display strong and consistent hand preferences on this task.

Subjects were individually presented with a piece of poly-vinyl-chloride tube 6 cm in length and 1.5 cm in diameter with peanut butter smeared inside. To remove the food, subjects had to hold the tube in one hand and use the fingers of other hand to retrieve the peanut butter. The hand used to retrieve the food from inside the tube was recorded as left or right. Every instance where an individual inserted their fingers into the tube, retrieved peanut butter and brought that hand to the mouth was recorded. Data were recorded until the subject lost interest in the tube as indicated by discarding the tube for at least 10 s. Each subject was tested four times with the task. Subjects performed a mean of 102 responses (*SE*±19.04) and showed high consistency in hand use across the four trials.

### Data analysis

To statistically adjust CC data for total brain volume, we followed a recommendation by Smith [36] wherein the square root of the CC area was divided by the cube root of total brain volume for each individual to bring all measures into the same geometric dimensionality. Additionally, we applied this adjustment to the various subdivisions of the CC. Where data did not violate assumptions of normality parametric statistics were employed; otherwise, nonparametric statistics were used.

Handedness index (HI) scores were determined for each subject by using the hand preference formula (#R−#L)/(#R+#L). The mean handedness index (*M*HI) was calculated by taking the average HI of all trials for each individual. *Z*-scores were calculated for *M*HI to determine if individuals displayed significant hand preferences and to classify subjects as right-handed, left-handed, or ambidextrous. Subjects with *z*-scores greater than 1.95 or less than −1.95 were classified as unambiguously right- or left-handed. Subjects with *z*-scores between 1.95 and −1.95 were classified as having no hand preference.

## Results

Individual area measurements of the CC, its subdivisions, total brain volume, body weights, *M*HI values for the tube task, and classification into dextral group are displayed in Tables 1 and 2. Table 1 displays the unadjusted measurements whereas Table 2 displays the adjusted CC measures. There was a significant correlation between overall CC area and total brain volume, *r* (18) = .51, *p* = .03.

Juveniles (*M* = 1.84±.06) did not have a significantly different CC:brain ratio than adults (*M* = 2.01±.17) [Mann Whitney *U* test, *z* = −1.65, *p* = .10, two-tailed]. However, as age positively correlated with the ratio of CC: total brain volume, *r* (18) = .57, *p* = .01, further analyses were conducted on the adult subjects only.

**Figure 2:**
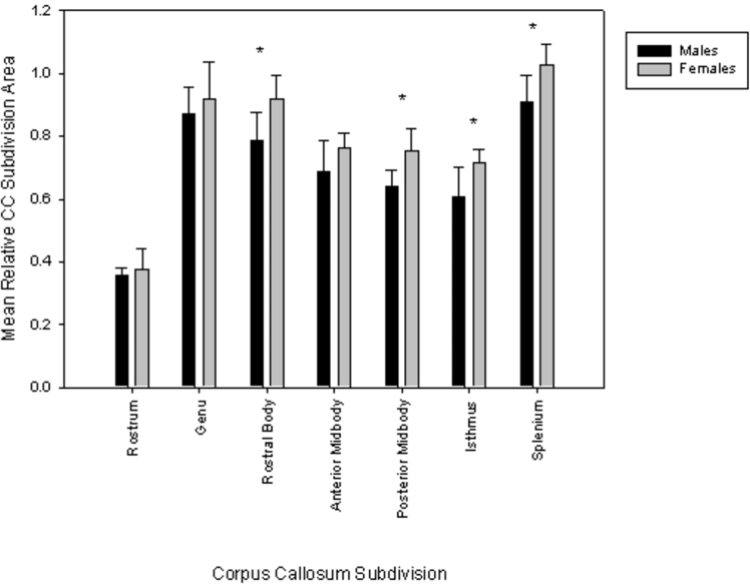
Mean (±SD) midsagittal area measures of the CC and its subdivisions as a function of sex.

Adult males (*M* = 1.86±.11) and adult females (*M* = 2.13±.09) differed significantly in overall CC:brain ratio [independent samples *t*-test, *t*(12) = −4.91, *p*<.001). An analysis of variance with sex as the between-subjects factor revealed significant sex differences for the CC subdivisions of rostral body [*F*(1, 12) = 9.14, *p* = .01, η^2^  = .43], posterior midbody [*F*(1, 12) = 11.51, *p*  = .005, η^2^  = .49], isthmus [*F*(1, 12) = 8.39, *p*  = .013, η^2^  = .41], and splenium [*F*(1, 12) = 8.57, *p* = .013, η^2^ = .41]. For all of these subdivisions females had larger areas than males [rostral body: female *M* = .92±.08, male *M* = .78±.09; posterior midbody: female *M* = .75±.07, male *M* = .64±.05; isthmus: female *M* = .72±.04, male *M* = .61±.09; splenium: female *M* = 1.03±.07, male *M* = .91±.09] (see Figure 2).

**Figure 3:**
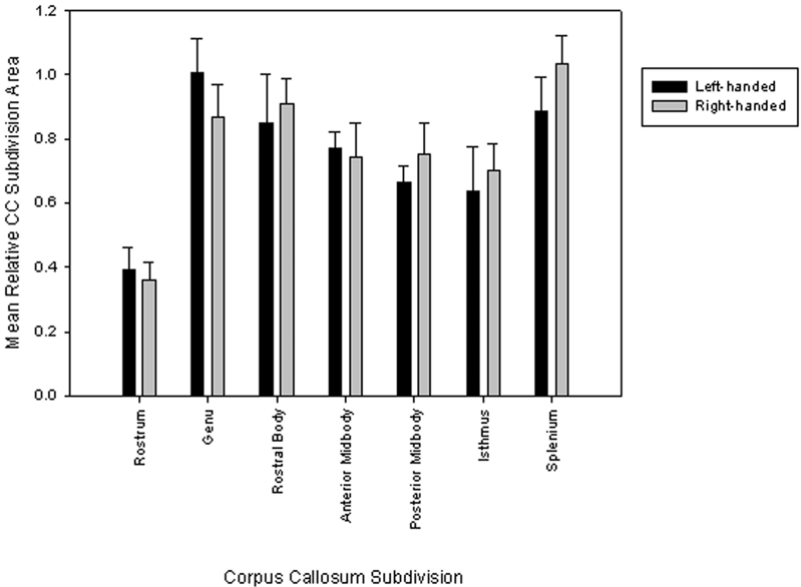
Mean (±SD) midsagittal area measures of the CC and its subdivisions as a function of hand preference.

An analysis of variance with handedness classification as the between-subjects factor indicated borderline significant effects of handedness on the genu [*F*(1,7) = 4.02, *p* = .09; η^2^ = .68] and splenium [*F*(1,7) = 4.95, *p* = .06; η^2^  = .41]. Left-handed individuals (*M* = 1.01±.11) had a larger genu than did right-handed individuals (*M* = .87±.10); right-handed individuals (*M* = 1.03±.09) had a larger splenium than left-handed individuals (*M* = .89±.10) (see Figure 3). Strength of hand preference (as measured by the *M*HI) and cc:brain ratio were not correlated, *r* (13) = .19, *p*  = .53.

A multifactorial analysis of variance with sex and handedness classification as the between-subjects factors revealed a significant interaction for the anterior midbody [*F*(1, 5) = 18.82, *p* = .007, η^2^ = .57], and a borderline significant effect was found for the posterior midbody [*F*(1,5) = 5.40, *p*  = .07, η^2^ = .29]. Right-handed males had a significantly smaller ratio in the anterior midbody than did right-handed females. No differences were found between left-handed males and left-handed females.

## Discussion

Several important findings emerged from our study. First, adult female capuchins have a significantly larger overall CC:brain ratio, rostral body, posterior midbody, isthmus and splenium than adult males. Second, we found borderline significant effects of handedness on corpus callosum morphology, with left-handed individuals having a larger relative genu and right-handed individuals having a larger splenium. Finally, a significant sex and handedness interaction was found, with right-handed capuchin males having a smaller anterior midbody than right-handed females. To our knowledge, this is the first demonstration of an interaction between CC morphology, sex and handedness in a nonhuman primate species.

Spatial-ability differences in males and females have been proposed to explain differences in the shape and size of the corpus callosum in humans, particularly in the posterior regions of the isthmus and splenium [2], [37], as this region connects areas of the parietal lobes known to be involved in spatial tasks. In support of this interpretation, Schoenemann [38] reported that women with smaller splenia scored better on a task of spatial ability. To our knowledge, whether or not capuchins show sex differences in spatial ability has not yet been demonstrated. It is certain, however, that capuchins rely heavily on processing complex visuospatial information. In the wild, *Cebus* monkeys utilize both arboreal and terrestrial substrates in their locomotor repertoire [39]. Capuchins are also noted for being very adept at capturing small rapid prey, such as birds, lizards, squirrels, and coatis [40]. Further behavioral data concerning sex differences in spatial abilities in the context of foraging and locomotion would clearly enhance our understanding of the functional significance of morphological sex differences of the corpus callosum.

The relationship between the direction of hand preference and CC morphology is not consistent across primate taxa. In humans, numerous studies have consistently found that left-handed and ambidextrous individuals have a larger midsaggital area of the CC than right-handed individuals [7], [8], [16], [41]. A recent study of chimpanzees showed the opposite relationship, however, with left-handed individuals having smaller CC subdivisions than right-handed chimpanzees [11]. Dunham and Hopkins proposed two explanations to explain this pattern: 1) the different measures used to assess handedness in humans (typically questionnaires) and chimpanzees (observable behavior), or 2) differences in organization of the CC. Our results, which correspond to the pattern observed in humans and assessed handedness in capuchins with a coordinated bimanual task, would seem to provide support for the importance of organization of the CC in influencing handedness. While sex differences in fiber composition of the CC have not been found in humans, fiber density has been shown to vary across CC subdivisions [42]. Both thin and thick fibers show increased density toward the posterior midbody as well as the posterior pole of the CC. Increased density of axons in the splenium subserve integration of visual field information from the two hemispheres, while the large heavily-myelinated callosal fibers of the midbody connect homotopic somatosensory and motor areas. Similar to humans, macaque CC show increased density of fibers in the midbody [43]. The relationship between the fiber architecture of the corpus callosum, asymmetries, and handedness remain poorly understood. However, if the observed sexual dimorphism of capuchin CC is related to differences in the distribution and/or density of axons, then this may provide the foundation for sex differences in hemispheric lateralization.

Our results provide support of the role of handedness influences on corpus callosum morphology, and thus hemispheric specialization in capuchin monkeys. As right-handed male capuchins had a significantly smaller anterior midbody than did right-handed females, our results further support the importance of left-hemispheric specialization in skilled motor actions, as has been proposed by some [*e.g*., 21]. We speculate that the observed interaction of sex and handedness on CC morphology is related to hemispheric specialization for motor integration of visuospatial information in the context of complex feeding actions. This hypothesis is supported by our previous findings that human-like patterns of neuroanatomical asymmetry in motor processing areas are related to handedness in capuchin monkeys [19], [20].
